# Postpartum Visit Attendance Increases the Use of Modern Contraceptives

**DOI:** 10.1155/2016/2058127

**Published:** 2016-12-13

**Authors:** Saba W. Masho, Susan Cha, RaShel Charles, Elizabeth McGee, Nicole Karjane, Linda Hines, Susan G. Kornstein

**Affiliations:** ^1^Virginia Commonwealth University Institute of Women's Health, P.O. Box 980319, Richmond, VA 23298, USA; ^2^Division of Epidemiology, Department of Family Medicine and Population Health, Virginia Commonwealth University, 830 E. Main Street, P.O. Box 980212, Richmond, VA 23298, USA; ^3^Department of Obstetrics and Gynecology, Virginia Commonwealth University School of Medicine, P.O. Box 980034, Richmond, VA 23298, USA; ^4^Division of Reproductive Endocrinology & Infertility, Department of Obstetrics, Gynecology and Reproductive Sciences, The University of Vermont College of Medicine, Smith 410, Main Campus, 111 Colchester Avenue, Burlington, VT 05401, USA; ^5^Virginia Premier Health Plan, Inc., 600 E. Broad Street, 4th Floor, Suite 400, Richmond, VA 23219, USA; ^6^Department of Psychiatry, Virginia Commonwealth University School of Medicine, 1200 E. Broad Street, P.O. Box 980710, Richmond, VA 23298, USA

## Abstract

*Background.* Delays in postpartum contraceptive use may increase risk for unintended or rapid repeat pregnancies. The postpartum care visit (PPCV) is a good opportunity for women to discuss family planning options with their health care providers. This study examined the association between PPCV attendance and modern contraceptive use using data from a managed care organization.* Methods.* Claims and demographic and administrative data came from a nonprofit managed care organization in Virginia (2008–2012). Information on the most recent delivery for mothers with singleton births was analyzed (*N* = 24,619). Routine PPCV (yes, no) and modern contraceptive use were both dichotomized. Descriptive analyses provided percentages, frequencies, and means. Multiple logistic regression was conducted and ORs and 95% CIs were calculated.* Results.* More than half of the women did not attend their PPCV (50.8%) and 86.9% had no modern contraceptive use. After controlling for the effects of confounders, women with PPCV were 50% more likely to use modern contraceptive methods than women with no PPCV (OR = 1.50, 95% CI = 1.31, 1.72).* Conclusions.* These findings highlight the importance of PPCV in improving modern contraceptive use and guide health care policy in the effort of reducing unintended pregnancy rates.

## 1. Introduction

Unintended pregnancy is a major public health problem in the US. According to a recent study analyzing data from the National Survey of Family Growth, in 2008, over half (51%) of the 6.6 million pregnancies in the US were unintended [1]. Additionally, a report using data from the National Survey of Family Growth that compared the proportion of US unintended pregnancies between 1982 and 2006–2010 found no significant improvements in the rates of unintended pregnancies over this extended time period [2]. Moreover, despite the availability of effective modern contraceptive methods, the rate of unintended pregnancies increased from 48% in 2001 to 51% in 2008 [1].

Unintended pregnancies are major public health concerns with potential detrimental effects on the health and wellbeing of infants, mothers, and society as a whole. Unintended pregnancy is associated with delayed prenatal care, smoking or drinking during pregnancy, preterm birth and low birth weight, poor attitudes towards parenting, poor infant development, and poor mother-infant relationships [3–6]. These levy a heavy burden on both state and federal economies. For instance, in 2010, total government expenditures on unintended pregnancies amounted to $21.0 billion, accounting for more than half of the $40.8 billion spent on all publicly funded pregnancies in 2010 [7].

Most women and couples want to have control over the timing and spacing of their childbearing for both economic and social reasons [8, 9]. The postpartum care visit (PPCV) is a good opportunity for patients and their significant others to have important conversations about family planning with their health care providers. The PPCV also allows providers to give necessary counseling and resources to women. Fundamentally, the best way of preventing unintended pregnancies is to provide women with an effective modern contraceptive method, which can be done through contraceptive counseling at a routine PPCV.

Delays in postpartum contraceptive use create a risky environment for women to have unintended or rapid repeat pregnancies [10, 11]. Although ovulation can occur within six weeks after delivery, it can occur as early as four weeks in nonbreastfeeding women [11]. Therefore, providing women with modern contraceptive methods shortly after they give birth is an effective way of curtailing the risk of an unintended pregnancy.

Some studies have shown the beneficial impact of PPCV attendance on greater contraceptive use [12–14] and reduced likelihood of rapid repeat pregnancy [15, 16] in select subgroups of women. Even women with no insurance or Medicaid were found to increase their use of an effective method after delivery if they attended counseling sessions [14]. However, the results did not account for important factors that could have affected postnatal services such as substance use or mental health problems (e.g., depression). Moreover, receipt of counseling was based on self-report data and did not necessarily indicate PPCV compliance or attendance. The fact remains that the PPCV is part of the standards of care for postpartum women and presents a suitable opportunity for contraceptive counseling. Strategies designed to reduce high rates of unintended pregnancies would benefit from targeting the postpartum period since women's opinions of subsequent pregnancies change over time [10].

The purpose of this study is to examine the association between PPCV attendance and the use of modern contraceptives among women who received care from a nonprofit managed care organization in the state of Virginia. These findings could provide a better understanding of the influence of the PPCV on modern contraceptive use and guide health care policy in the effort of reducing unintended pregnancy rates.

## 2. Materials and Methods

Data came from Virginia Premier, a managed care organization that coordinates health care services for low-income individuals enrolled in Virginia Medicaid. Claims and demographic and administrative information were available for women with singleton births between the years of 2008 to 2012. Information on the most recent birth was analyzed for mothers who gave birth to more than one infant during the study period. Thus, the total sample size was comprised of 24,619 women. This study was approved by the Virginia Commonwealth University Institutional Review Board.

The demographic dataset included information on maternal characteristics such as age, race, and region of residence. The birth event dataset included birthing information, such as delivery date, delivery type (vaginal, C-section), gestational age, and birth weight of infant, as well as NICU status and length of stay. Medical claims data were also included and provided the International Classification of Diseases, Ninth Revision (ICD-9) codes used to determine postpartum visit attendance, pregnancy complications, and substance abuse. Additionally, interview data was collected by case managers during both prenatal and postpartum period for clinical administrative purposes. These consisted of personal information such as education level, primary language spoken, smoking status, alcohol use, breastfeeding intention during pregnancy and actual feeding method (i.e., breastfeeding, bottle feeding, or both), depression, and birth control use, as well as the instances and types of case management.

The exposure of interest, PPCV attendance, came from medical claims data containing ICD-9-Clinical Modification (ICD-9-CM) for medical diagnoses and procedures on claims for services. Postpartum care and evaluation at the follow-up visit were determined by an ICD-9 code for routine postpartum follow-up (V24.2). This information was categorized as “yes” or “no.”

The outcome, modern contraceptive use, came from the interview data which were collected by case managers for clinical administrative purposes. Women who reported using any modern contraceptive methods such as “birth control pill,” “Depo-Provera,” “Norplant,” “patch,” “ring,” “IUD” (i.e., intrauterine device), “condoms and foam,” and “diaphragm” were classified as “users.” Those who did not indicate the use of any methods were considered “nonusers.”

Sociodemographic factors included maternal age (≤20 years; 21–29 years; ≥30 years), race/ethnicity (White; Black; Hispanic; other), and the highest educational level (less than high school; high school graduate; greater than high school). Maternal region of residence in Virginia was categorized into seven regions: Danville/Lynchburg, Far Southwest, Fredericksburg, Richmond, Roanoke, Tidewater, and Western. Location of the majority of medical services was defined as the type of health care system most utilized by each individual patient (private office; hospital; health department; or federally qualified health centers (FQHC)). This was based on the total number of visits to each health care setting calculated for each woman. Substance use and mental health problems included tobacco use disorder (yes, no), drug abuse/dependence (yes, no), alcohol abuse/dependence (yes, no), and history of depression (yes, no). Pregnancy complications including preeclampsia, eclampsia, hypertension, diabetes, anemia, cervical incompetence, ectopic pregnancy, uterine inertia, premature separation of placenta, and placenta previa (yes; no), type of delivery (normal vaginal, caesarean section), and birth outcomes (normal weight and term; normal weight and preterm; low birth weight and term; low birth weight and preterm), where preterm birth was defined as gestational age of <37 weeks and low birth weight was defined as <2500 grams, were also assessed.

Descriptive analyses were conducted and percentages, frequencies, and means were reported. Bivariate analysis was conducted to examine factors associated with attending a PPCV or modern contraceptive use. To adjust for potential confounders, multivariable logistic regression was conducted and odds ratios (OR) and 95% confidence intervals (CIs) were calculated. Potential confounders were identified and included in the model if the variable resulted in a 10% or greater change in the estimate.

## 3. Results

The average age of the study population was 24.9 (standard deviation = 5.3) years. The majority of the women were 21–29 years of age (59.8%), had high school education (51.4), used hospitals for the majority of their medical services (87.3%), had normal vaginal deliveries (67.8**%**), and delivered their babies at normal weight and term (86.7%) (Table 1). Nearly half (49.3%) of the women attended their postpartum visit and 86.9% had no recorded modern contraceptive use.

Factors associated with modern contraceptive use included sociodemographic factors (i.e., age, race, location of majority of services, and region of residence in Virginia; *p* < 0.0001), health behavioral factors (i.e., tobacco use, *p* = 0.0010; drug abuse/dependence, *p* = 0.0140), and pregnancy complications (*p* = 0.0202). Specifically, modern contraceptive users included a greater proportion of women who were aged 20 years or younger and Black or Hispanic. Importantly, a greater proportion of modern contraceptive users attended their PPCV (57.1%) compared to nonusers (48.1%, *p* < 0.0001). Moreover, there was a significant difference in the distribution of region of residence for women who used modern contraceptives and those who did not. Specifically, a greater proportion of women who used contraception resided in highly populated urban regions than women who did not use contraception (Richmond, 17.4% versus 14.3%; Roanoke, 31.1% versus 28.8%). A greater proportion of nonusers resided in regions with smaller populations than women who used contraception (Danville/Lynchburg, 10.3% versus 8.8%; Fredericksburg, 6.8% versus 6.1%).

More than half of the women who were ≤20 years of age (50.0%), Black (50.9%), and highly educated (54.9%) attended their postpartum visits (Table 2). Women who received most of their medical services from a hospital or health department/FQHC had significantly increased odds of attending their postpartum visits than women who utilized mostly health services from private offices (COR [crude odds ratio] = 1.36, 95% CI = 1.24–1.50; COR = 1.99, 95% CI = 1.72–2.30, resp.). Likewise, compared to women from Fredericksburg, those who lived in areas with greater poverty were more likely to attend their postpartum visit, for example, Danville/Lynchburg, Western, and Richmond. In fact, women from Danville/Lynchburg were nearly three times as likely to attend postpartum visits (COR = 3.32, 95% CI = 2.92–3.79). Additionally, women with a history of depression were more likely to attend their postpartum visits when compared with women with no history of depression (COR = 1.13, 95% CI = 1.02–1.24). In terms of health behavioral factors, tobacco users and those diagnosed with drug abuse/dependence were less likely to attend their postpartum visits than nonusers and women not diagnosed with drug abuse/dependence (OR = 0.83, 95% CI = 0.78–0.88; OR = 0.67, 95% CI = 0.60–0.74, resp.). Women who experienced pregnancy complications had 1.23 times the odds of attending their postpartum visit compared with women with no complications during pregnancy (COR = 1.23, 95% CI = 1.17–1.30). There were no significant differences in postpartum visit attendance between women with normal vaginal delivery and women with C-sections. Moreover, when considering birth outcomes, women who delivered infants that were both of low birth weight and preterm were significantly less likely to attend their postpartum visit than women who delivered infants that were of normal weight and term (Table 2).

A significant association between postpartum visit attendance and modern contraceptive use was observed (Table 3). Women who attended their postpartum visit were 44% more likely to use postpartum contraception compared to women with no postpartum visit (OR = 1.44, 95% CI = 1.33–1.55). No covariate changed the estimate by 10% or greater; therefore, the unadjusted model was retained. Nonetheless, estimates remained robust and statistically significant even after controlling for age, race, education, location of majority of services, region of residence, tobacco use, drug abuse/dependence, alcohol abuse/dependence, history of depression, pregnancy complications, method of delivery, and birth outcomes (AOR [adjusted odds ratio] = 1.50, 95% CI = 1.31–1.72). In other words, women attending postpartum visits were 50% more likely to use modern contraceptive methods than women who did not attend their postpartum visit (Table 3).

## 4. Discussion

Despite the majority of the study population having no postpartum modern contraceptive use, women were 50% more likely to use contraception after delivery if they attended a postpartum appointment compared to those who did not attend their PPCV. This was independent of sociodemographic factors, substance use, depression, and pregnancy or birth complications.

Findings from the current study suggest that the period following childbirth is a crucial and opportune time for new mothers receiving publicly funded health care services to get insurance coverage for contraception and counseling and guidance on effective methods to avoid unintended and rapid repeat pregnancy [17, 18]. For example, Thiel de Bocanegra et al. examined health records for Medicaid recipients in California (*n* = 117,644) and reported that although only 41% had a modern contraceptive claim within 90 days of giving birth, receipt of contraception at the first postpartum clinic visit was significantly associated with avoiding another pregnancy within 6 and 18 months of a previous live birth (AOR = 1.63, 95% CI = 1.49, 1.80; AOR = 1.57, 95% CI = 1.50, 1.65, resp.) [17]. Recognizing the importance of postpartum care, the American Academy of Pediatrics and the American College of Obstetricians and Gynecologists recommend that new mothers have a checkup four to six weeks after delivery [19].

Extant literature supports the results from our study in that patients who do not attend their PPCV are less likely to use contraception or effective methods (e.g., long-acting reversible contraception or LARC) [12–17, 20, 21]. For instance, DePiñeres et al. examined factors associated with postpartum contraception using self-report data from New Mexico PRAMS (1998-1999). Women aged 35 years or more, unmarried and lacking a postpartum visit, had increased risk of no postpartum contraception [21]. Specifically, the odds of postpartum modern contraceptive use were nearly threefold greater in women who reported attending their PPCV than in those who did not attend (AOR = 3.06, 95% CI = 2.17, 4.31) [21]. Likewise, a recent study that assessed women's barriers to receiving LARC in the postpartum period reported common reasons for nonuse being having to come back for another insertion visit (45%) and being unable to afford LARC methods (11%) [20]. Moreover, women who were interested in but not using LARC were more likely to have missed their postpartum visit compared to women using effective methods (*p* = 0.001) [20]. This can be especially problematic when nearly half of the women resume sexual intercourse within six weeks of giving birth, regardless of lactation or delivery method [18]. As such, a delay in effective modern contraceptive method initiation can be detrimental for women of low income or with high-risk pregnancies.

Focused contraceptive counseling and education by health providers is essential to improve women's reproductive health and postnatal care. In a qualitative study comprised of postpartum, urban, and minority women, participants showed preference for frequent contraceptive counseling sessions throughout pregnancy, with reinforcement and reevaluation of decisions after delivery [22]. Thus, public health strategies seeking to reduce high rates of unintended pregnancies should include the postpartum period since women's opinions of subsequent pregnancies change over time [10] and the PPCV is already an important standard of care for postpartum women.

This study was strengthened by the use of claims data rather than self-report data to ensure more objective measures of key variables of interest such as contraception and PPCV. We also considered a myriad of factors that could affect women's use of contraception or attendance such as sociodemographic characteristics, health behaviors, history of depression, and pregnancy/birth outcomes. The focus on a high-risk population receiving publicly funded health care services can provide more useful information on areas to improve in health care delivery and intervention efforts. Nonetheless, there were some study limitations. The dataset did not contain information on the quality of patient-provider interaction during PPCVs (e.g., topics covered, duration of visit, and communication style) that would be better assessed in qualitative studies [23]. Additionally, confounding factors such as breastfeeding were not assessed due to lack of complete data. We were also unable to ascertain whether certain modern contraceptive methods that were claimed under the insurance (e.g., birth control pills, barrier methods) were actually used.

## 5. Conclusions

In conclusion, among women with Medicaid, those who attended a postpartum visit were 50% more likely to use a modern contraceptive method after delivery. The postpartum visit is an apt setting for health providers to educate women on family planning options to ensure proper birth spacing and prevent unintended or rapid repeat pregnancies. Reducing barriers for access to and use of PPCV is greatly needed in low-income women or other vulnerable populations who face additional challenges with unstable housing, transportation barriers, and language barriers [24]. Future studies are needed to evaluate effective components of care (e.g., multiple counseling sessions) and patient-provider communication.

## Figures and Tables

**Table 1:** Distribution of population characteristics by postpartum contraceptive use.

	Contraceptive use		Total population *N* = 24,619	*χ* ^2^	*p* value
	Yes *N* = 3,232	No *N* = 21,387			
	Column %				
Age				84.43	<0.0001
≤20 years	26.1	21.6	22.2		
21–29 years	61.2	59.6	59.8		
≥30 years	12.7	18.8	18.0		
Race				52.49	<0.0001
White	51.6	56.2	55.7		
Black	28.3	25.1	25.5		
Hispanic	5.6	3.0	3.3		
Other	14.5	15.7	15.5		
Education				0.90	0.6388
<High school	19.2	19.4	19.3		
High school	50.9	51.8	51.4		
>High school	29.9	28.8	29.3		
Location of majority of services				23.32	<0.0001
Private	5.9	7.9	7.7		
Hospital	89.9	86.9	87.3		
Health department/FQHC	4.3	5.2	5.1		
Region of residence in Virginia				107.36	<0.0001
Danville/Lynchburg	8.8	10.3	10.1		
Far Southwest	0.9	3.9	3.5		
Fredericksburg	6.1	6.8	6.7		
Richmond	17.4	14.3	14.7		
Roanoke	31.1	28.8	29.1		
Tidewater	17.4	18.6	18.4		
Western	18.3	17.4	17.5		
Tobacco use	24.7	27.5	27.1	10.88	0.0010
Drug abuse/dependence	5.0	6.1	6.0	6.04	0.0140
Alcohol abuse/dependence	1.0	1.0	1.0	0.08	0.7831
History of depression	7.3	6.6	6.7	2.12	0.1454
Pregnancy complications	42.4	40.3	40.6	5.39	0.0202
Delivery				2.11	0.1463
Normal vaginal	68.9	67.6	67.8		
C-section	31.1	32.4	32.2		
Birth outcomes				6.79	0.0787
Normal weight & term	88.1	86.5	86.7		
Normal weight & preterm	3.3	3.9	3.9		
Low birth weight & term	3.2	3.6	3.6		
Low birth weight & preterm	5.5	6.0	5.9		
Postpartum visit attendance				91.28	<0.0001
Yes	57.1	48.1	49.3		
No	42.9	51.9	50.8		

FQHC: federally qualified health centers; g: grams; wks: weeks. Normal weight: ≥2500 grams; low birth weight: <2500 grams; term: ≥37 weeks; preterm: <37 weeks.

**Table 2:** Factors associated with postpartum visit attendance.

	Postpartum visit (row %)	Crude OR (95% CI)
Age		
≤20 years	50.0	1.00
21–29 years	49.4	0.97 (0.92–1.04)
≥30 years	48.0	0.92 (0.85–1.00)
Race		
White	49.6	1.00
Black	50.9	1.05 (0.98–1.13)
Hispanic	48.7	0.97 (0.82–1.16)
Other	48.9	0.97 (0.88–1.05)
Education		
<High school	51.8	0.88 (0.76–1.03)
High school	54.3	0.98 (0.87–1.10)
>High school	54.9	1.00
Location of majority of services		
Private	41.9	1.00
Hospital	49.5	1.36 (1.24–1.50)^*∗*^
Health department/FQHC	58.9	1.99 (1.72–2.30)^*∗*^
Region of residence in Virginia		
Danville/Lynchburg	70.2	3.32 (2.92–3.79)^*∗*^
Far Southwest	48.6	1.33 (1.13–1.57)^*∗*^
Fredericksburg	41.5	1.00
Richmond	49.4	1.38 (1.23–1.55)^*∗*^
Roanoke	45.0	1.16 (1.04–1.29)
Tidewater	42.8	1.05 (0.94–1.18)
Western	54.1	1.66 (1.48–1.86)
Tobacco use		
No	50.5	1.00
Yes	45.8	0.83 (0.78–0.88)
Drug abuse/dependence		
No	49.9	1.00
Yes	39.9	0.67 (0.60–0.74)
Alcohol abuse/dependence		
No	49.3	1.00
Yes	44.7	0.83 (0.65–1.07)
History of depression		
No	49.1	1.00
Yes	52.0	1.13 (1.02–1.24)
Pregnancy complications		
No	47.2	1.00
Yes	52.3	1.23 (1.17–1.30)
Delivery		
Normal vaginal	49.3	1.00
C-section	49.4	1.00 (0.95–1.06)
Birth outcomes		
Normal weight & term	49.6	1.00
Normal weight & preterm	49.8	1.01 (0.89–1.15)
Low birth weight & term	46.8	0.89 (0.78–1.02)
Low birth weight & preterm	45.7	0.86 (0.77–0.95)

OR: odds ratio; CI: confidence interval; FQHC: federally qualified health centers; g: grams; wks: weeks. Normal weight: ≥2500 grams; low birth weight: <2500 grams; term: ≥37 weeks; preterm: <37 weeks. ^*∗*^ *p* < 0.05.

**Table 3:** Association between postpartum visit attendance and contraceptive use.

	^a^OR (95% CI)	^b^OR (95% CI)
Postpartum visit	^*∗*^1.44 (1.33–1.55)	^*∗*^1.50 (1.31–1.72)
No postpartum visit	1.00	1.00

OR: odds ratio; CI: confidence interval.

^a^No factor changed the estimate by 10% or greater.

^b^Fully adjusted model controlling for age, race, education, location of majority of services, region of residence, tobacco use, drug abuse/dependence, alcohol abuse/dependence, history of depression, pregnancy complications, delivery, and birth outcomes.

^*∗*^Statistically significant.
